# Protective Effects of Mackerel Protein Hydrolysates Against Oxidative Stress-Induced Atrophy in C2C12 Myotubes

**DOI:** 10.3390/foods14142430

**Published:** 2025-07-10

**Authors:** Gyu-Hyeon Park, Syng-Ook Lee

**Affiliations:** Department of Food Science and Technology, Keimyung University, Daegu 42601, Republic of Korea; soulpkh@naver.com

**Keywords:** sarcopenia, myotubes, oxidative stress, mackerel, protein hydrolysates, alcalase

## Abstract

Muscle aging and atrophy in the elderly are closely associated with increased oxidative stress in muscle tissue. Bioactive peptides derived from protein hydrolysates have emerged as promising functional ingredients for alleviating sarcopenia due to their antioxidant properties and enrichment in essential amino acids. In a preliminary screening, mackerel protein hydrolysate (MPH) showed notable protective effects in a myotube atrophy model. This study evaluated the anti-atrophic potential of MPHs produced using different enzymes in H_2_O_2_-treated C2C12 myotubes. Among five hydrolysates, the alcalase-derived hydrolysate (MHA) demonstrated the most potent effects in maintaining myotube diameter, restoring myosin heavy chain (MYH) expression, and downregulating the atrophy-related genes MAFbx and MuRF1. Mechanistically, MHA activated the Akt/FoxO signaling pathway and inhibited NF-κB activation, thereby reducing muscle protein degradation. Additionally, MHA significantly lowered intracellular ROS levels and showed strong direct antioxidant activity. Amino acid and molecular weight profiling revealed high levels of essential amino acids and low-molecular-weight peptides, suggesting a synergistic contribution to its bioactivity. These findings suggest that MHA is a promising food-derived functional material with anti-atrophic and antioxidant properties and may be useful in preventing or managing age-related muscle loss such as sarcopenia, warranting further preclinical validation.

## 1. Introduction

According to the United Nations’ World Population Ageing 2023 report, individuals aged 65 and older represented approximately 12% of the global population in 2023, and this proportion is projected to rise to around 16% by 2050 [1]. This demographic shift has significant implications for public health, particularly concerning age-related diseases such as sarcopenia, which is characterized by the progressive loss of muscle mass and strength [2]. Sarcopenia, a progressive loss of skeletal muscle mass and strength, is a key age-related condition that severely impairs mobility and quality of life. It is also a major risk factor for falls, fractures, and increased mortality, making its prevention and treatment a critical area of research [3,4]. The global prevalence of sarcopenia varies depending on factors such as age, sex, body composition, and diagnostic criteria, with recent estimates suggesting that approximately 10–16% of older adults are affected [5].

One of the key mechanisms contributing to sarcopenia is the imbalance in muscle protein metabolism, driven by chronic inflammation and oxidative stress associated with aging [6,7]. Muscle protein metabolism is primarily regulated by a balance between protein synthesis and degradation. The synthesis pathway is controlled by the Akt/mTOR signaling cascade, while the degradation pathway is activated through the ubiquitin-proteasome system. The ubiquitin-proteasome system is regulated by transcription factors such as FoxO and NF-κB, which induce the expression of ubiquitin E3 ligases, including MAFbx and MuRF1, thereby facilitating proteasomal degradation of muscle proteins [8,9].

To date, several food-derived compounds, such as *Schisandrae chinensis* extract [10], turmeric extract [11], low-molecular-weight whey protein hydrolysate [12], and fermented oyster extract [13], have been reported to mitigate muscle aging and atrophy. However, the range of available functional food ingredients targeting sarcopenia remains limited, underscoring the need for new dietary strategies to support muscle health.

Among various contributing factors, oxidative stress plays a central role in muscle atrophy and aging-related muscle loss. Recently, bioactive peptides derived from protein hydrolysates have gained attention due to their ability to provide essential amino acids required for muscle synthesis while also exhibiting potent antioxidant activity [14,15]. For example, whey protein hydrolysates and their peptide fractions have been reported to improve sarcopenia [16], supporting the notion that protein hydrolysates containing bioactive peptides may serve as protective agents against muscle atrophy.

Based on this, we hypothesized that food-derived protein hydrolysates could help mitigate aging-related muscle atrophy. In preliminary screenings, we enzymatically prepared protein hydrolysates from various animal-derived food sources and evaluated their effects in a cell-based model. Among the tested hydrolysates, mackerel (*Scomber japonicus*) protein hydrolysate showed the most promising protective activity against H_2_O_2_-induced atrophy in C2C12 myotubes. Mackerel, a protein- and omega-3-rich food source, has been reported to possess various physiological benefits, including antioxidant, antihypertensive, anti-fatigue, and bone-supporting activities [17,18,19]. However, its potential for muscle aging or sarcopenia intervention remains underexplored.

Therefore, the present study aimed to (1) develop a mackerel-derived protein hydrolysate using food-grade enzymatic hydrolysis, (2) evaluate its protective effects against oxidative stress-induced myotube atrophy in C2C12 cells, and (3) elucidate the underlying molecular mechanisms contributing to its anti-atrophic activity.

## 2. Materials and Methods

### 2.1. Materials

Alcalase, bromelain, flavourzyme, neutrase, and papain were purchased from Novo (Bagsvaerd, Denmark) via Daejong Sangsa (Seoul, Republic of Korea). The TNBS reagent for hydrolysis assessment was obtained from G-Bio-Sciences (St. Louis, MO, USA). DMEM high glucose, penicillin/streptomycin, fetal bovine serum (FBS), and PBS for C2C12 cell culture were sourced from Welgene (Gyeongsan, Republic of Korea). The MTT reagent for cell viability assays was acquired from Invitrogen (Carlsbad, CA, USA). RIPA lysis buffer was obtained from Thermo Fisher (Waltham, MA, USA), while the protein marker, ECL detection kit, and Hybond-P PVDF membrane were from Bio-Rad (Hercules, CA, USA). Phosphatase and protease inhibitors were purchased from GenDEPOT (Katy, TX, USA). PCR primers were synthesized by Bioneer (Daejeon, Republic of Korea), and TRIzol reagent for RNA extraction was obtained from Thermo Fisher. Other analytical reagents and solvents were purchased from Sigma-Aldrich (St. Louis, MO, USA).

### 2.2. Preparation of Mackerel Protein Hydrolysates

Mackerel (*Scomber japonicus*) powder was obtained from Taemyung Food Co., Ltd. (Seoul, Republic of Korea) and stored at −20 °C until use. Mackerel powder was prepared by removing the head, viscera, and bones from mackerel, followed by thorough washing. The flesh was steamed for 20 min, dried at 65 °C for 16 h using a hot-air dryer, and then ground and sieved (≤80 mesh). The mackerel powder (moisture content: 6.77 ± 0.21%) was mixed with triple-distilled water to prepare a 4% (*w*/*v*) substrate solution, which was then incubated in a water bath at 90 °C for 20 min to inactivate endogenous enzymes. The substrate solution was treated with 1% (*w*/*w*) of each protein-hydrolyzing enzyme: alcalase (≥2.4 Anson Units/g), bromelain (1200 Gelatin-Digesting Units/g), flavourzyme (500 Leucine Amino Peptidase Units/g), neutrase (8 KiloNovo Pretease Units/g), and papain (≥30 N-α-benzyoyl-L-arginine ethyl ester Units/mg), and hydrolyzed at 55 °C, 130 rpm for 12 h in a shaking incubator (IST-4075, Jeio Tech Co., Ltd., Daejeon, Republic of Korea). The enzymatic reaction was then terminated by heating at 90 °C for 20 min. After cooling, the hydrolysates were centrifuged at 13,000× *g* for 20 min, and the supernatant was sequentially filtered using a cell strainer and a 0.22 μm membrane filter. The filtered supernatant was then freeze-dried for 72 h, and the resulting mackerel protein hydrolysates (MPH) were stored at −80 °C until further use.

### 2.3. Determination of Degree of Hydrolysis by TNBS Assay

The degree of hydrolysis (DH) of MPH was analyzed using the TNBS assay, as described by Cho and Lee (2020) [15]. Hydrolysates collected at different time intervals were centrifuged at 3000× *g*, 4 °C for 10 min, and the supernatant was used for the assay. A 0.01% TNBS solution was prepared by mixing 100 mM sodium bicarbonate (pH 8.5) and TNBS (*v*/*v*). A 50 μL aliquot of TNBS solution was added to 100 μL of each hydrolysate sample, followed by incubation at 37 °C for 2 h. The reaction was stopped by adding 50 μL of 10% SDS and 25 μL of 1N HCl, and the absorbance was measured at 335 nm using a microplate spectrophotometer (Epoch, Biotek Instruments, Winooski, VT, USA). Tyrosine was used as a standard for calculating DH.

### 2.4. Determination of Protein Molecular Weight Patterns by SDS-PAGE

SDS-PAGE was performed to analyze the molecular weight distribution of MPH hydrolyzed by different enzymes. A 15% polyacrylamide gel was prepared, and each hydrolysate (10 μg of protein) was loaded onto the gel. A molecular weight marker (Precision Plus Protein Dual Color Standards, Cat. #1610374, Bio-Rad, Carlsbad, CA, USA) was used for reference. The gel was stained with 0.1% Coomassie Brilliant Blue and destained using an acetic acid:methanol:water (1:3:6) solution. Images of the gels were captured using the Gel Logic 2200 PRO Imaging System (Carestream Health Inc., Rochester, NY, USA).

### 2.5. C2C12 Cell Culture, Differentiation, and Myotube Atrophy Induction

C2C12 cells were cultured in DMEM high glucose supplemented with 10% FBS and 1% penicillin/streptomycin in a 5% CO_2_ incubator at 37 °C. For differentiation, cells were seeded in 6-well plates at a density of 1.5 × 10^5^ cells/well. Upon reaching 100% confluence, the medium was replaced with differentiation medium (DMEM high glucose containing 2% FBS and 1% penicillin/streptomycin), and cells were incubated for 6 days, with media changes every 2 days to induce myotube formation. To induce myotube atrophy, 2 mM H_2_O_2_ was added for 24 h, while MPH (25–200 μg/mL) was pre-treated 1 h prior to H_2_O_2_ exposure to evaluate its protective effects.

### 2.6. Measurement of Myotube Diameter

Differentiated C2C12 cells were pre-treated with MPH (25–200 μg/mL) for 1 h, followed by treatment with 2 mM H_2_O_2_ for 24 h. Images were captured at 100× magnification using a phase-contrast microscope (Leica Microsystems, Wetzlar, Germany), and myotube diameters were quantified using the i-Solution software (DT version). The average myotube diameter was calculated from 10 randomly selected myotubes.

### 2.7. Immunofluorescence Assay for Myosin Heavy Chain (MYH) Expression in C2C12 Myotubes

C2C12 cells were cultured and differentiated in 24-well plates with 12 mm glass coverslips. After differentiation, cells were pre-treated with the sample (25–200 μg/mL) for 1 h, followed by H_2_O_2_ treatment for 24 h to induce myotube atrophy. Coverslips were washed with PBS, fixed with 4% paraformaldehyde (in PBS) for 20 min, and washed again. Cells were then permeabilized with 0.3% Triton X-100 and blocked with 5% BSA for 1 h. After blocking, cells were incubated overnight at 4 °C with primary MYH antibody (Santa Cruz Biotechnology, 1:50 dilution), followed by Alexa Fluor^®^ 488-conjugated goat anti-mouse IgG H&L (Abcam, 1:200 dilution) for 2 h at 4 °C. After three PBS washes, nuclei were stained with 4′,6-diamidino-2-phenylindole (DAPI; Abcam, Cambridge, MA, USA). Fluorescence images were captured using a Zeiss Axioskop50 fluorescence microscope (Carl Zeiss, Oberkochen, Germany) equipped with a Jenoptik ProgResC5 Cool camera (Jena, Germany).

### 2.8. Amino Acid Composition Analysis

For amino acid composition analysis, 0.05 g of MPH was hydrolyzed with 1 mL of 6 N HCl under nitrogen flushing for 1 min and then incubated at 110 °C for 24 h. The sample was then dried at 80 °C for approximately 48 h, after which it was reconstituted with 1 mL of 0.02 N HCl and sonicated. The solution was filtered through a 0.45 μm membrane filter before injection. The amino acid composition was analyzed using an automatic amino acid analyzer (LA8080, Hitachi, NaKa, Japan). For free amino acid analysis, ethanol was added to 0.1 g of MPH, diluted 10 times, of which 1 mL was obtained, and an equal amount of 5% trichloroacetic acid was added, and the mixture was stirred. The stirred solution was centrifuged at 1000 rpm for 10 min, and only the supernatant was filtered using a 0.2 μm filter and injected. Amino acid analysis was performed using an automatic analyzer.

### 2.9. Statistical Analysis

All experimental results were expressed as mean ± standard deviation (SD) from at least three independent experiments. Student’s t-test (Sigma Plot Version 10.0; Systat Software Inc., San Jose, CA, USA) was used to compare differences between two groups. For comparisons involving three or more groups, one-way analysis of variance (ANOVA) was conducted using SPSS Version 27.0 (Statistical Package for the Social Sciences, SPSS Inc., Chicago, IL, USA), followed by Duncan’s multiple range test (*p* < 0.05) for post-hoc analysis.

All other Materials and Methods are described in the Supplemental Materials.

## 3. Results and Discussion

### 3.1. Characteristics of Mackerel Protein Hydrolysates Prepared Using Different Enzymes

To evaluate the characteristics of mackerel protein hydrolysates (MPHs) produced using different enzymes, five proteolytic enzymes (alcalase, flavourzyme, neutrase, bromelain, and papain) were used. The TNBS assay was conducted to measure the concentration of available amino groups at different time points, allowing for a comparison of hydrolysis efficiency among the enzymes. While an increase in free amino groups generally reflects greater protein breakdown, it is important to note that TNBS assay results are influenced not only by the extent of hydrolysis but also by the specific cleavage patterns, substrate affinity, and the mode of action of each enzyme [15,20,21].

**Figure 1:**
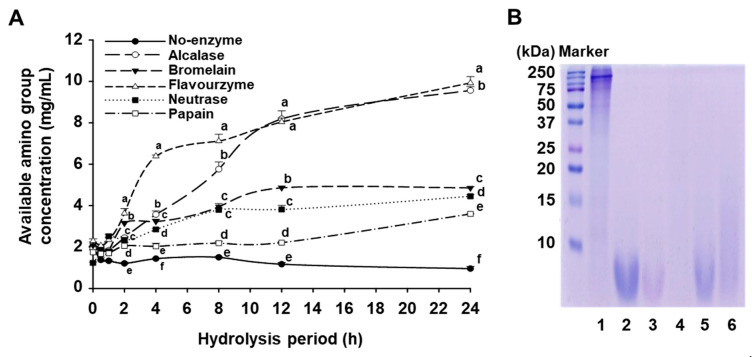
Hydrolysis characteristics of mackerel protein by various proteolytic enzymes. Mackerel powder was treated with each enzyme for 24 h at 55 °C. The available amino group concentration (**A**) and SDS-PAGE profiles (**B**) were determined, as described in the Section 2. (**B**) 1: No enzyme, 2: Alcalase, 3: Bromelain, 4: Flavourzyme, 5: Neutrase, 6: Papain. Data are presented as mean ± SD (n ≥ 3), and different superscripts (a–f) at the same time period are significantly different at *p* < 0.05 by Duncan’s multiple range test.

The hydrolysis degree, expressed as tyrosine content, was determined after 12 h of reaction. The tyrosine-equivalent available amino group concentrations for each enzyme treatment were as follows: 8.19 mg/mL for alcalase, 4.86 mg/mL for bromelain, 8.04 mg/mL for flavourzyme, 3.81 mg/mL for neutrase, 2.21 mg/mL for papain, and 1.17 mg/mL for the non-enzymatic control (Figure 1A). Notably, alcalase and flavourzyme exhibited a rapid increase in available amino groups during the initial 12 h, followed by a plateau. In contrast, neutrase, bromelain, and papain showed a gradual increase over 24 h, with papain displaying the lowest hydrolysis efficiency across all time points. Interestingly, while bromelain typically shows low hydrolysis efficiency for animal proteins [20,22,23], it exhibited comparable or slightly higher free amino group release than neutrase in mackerel protein hydrolysis. Since most enzymes showed no significant difference in hydrolysis between 12 and 24 h, and flavourzyme exhibited a continuous increase in hydrolysis up to 12 h, the optimal hydrolysis time for further analyses was set at 12 h.

To assess the molecular weight distribution of MPHs after 12 h of hydrolysis, SDS-PAGE was performed. Compared to non-enzymatic control, hydrolysates produced using alcalase, flavourzyme, and bromelain showed almost complete degradation of proteins >10 kDa, indicating effective enzymatic hydrolysis (Figure 1B). In contrast, neutrase and papain hydrolysates retained some proteins >10 kDa, aligning with their lower hydrolysis degree observed in the TNBS assay. Notably, flavourzyme hydrolysates contained fewer proteins even below 10 kDa, suggesting the production of smaller peptides compared to the other enzymes.

Based on the TNBS assay and SDS-PAGE results, the hydrolysis time was standardized at 12 h, and the yield of MPHs produced by each enzyme was measured (Supplemental Table S1). The highest yield was obtained from alcalase (47.18%), followed by bromelain (39.67%), flavourzyme (36.34%), neutrase (29.26%), and papain (19.43%), while the non-enzymatic control had the lowest yield at 12.25%. Previous studies on enzymatic hydrolysis of animal- and plant-derived proteins have reported yields ranging from 20% to over 40% [23,24]. The relatively high yield of MPHs observed in this study suggests that mackerel protein is a highly suitable substrate for enzymatic hydrolysis, particularly when processed with alcalase.

### 3.2. Alcalase-Derived Mackerel Hydrolysate (MHA) Protects Myotube Atrophy in H_2_O_2_-Treated C2C12 Myotubes

It is well established that H_2_O_2_ treatment in differentiated C2C12 myotubes induces atrophy via activation of the ubiquitin-proteasome pathway [25,26]. Prior to evaluating the protective effects of MPHs, we optimized the H_2_O_2_-induced myotube atrophy model. Based on our preliminary data, treatment with 2 mM H_2_O_2_ for 24 h was determined to be the optimal condition for inducing myotube atrophy. 

Although dexamethasone is frequently used to induce muscle atrophy in animal models, it functions as a glucocorticoid receptor agonist, mimicking cortisol-induced catabolic effects [25,27]. Since aging-related muscle atrophy is primarily driven by oxidative stress, the H_2_O_2_-induced model was deemed more physiologically relevant for studying sarcopenia-related muscle loss.

**Figure 2:**
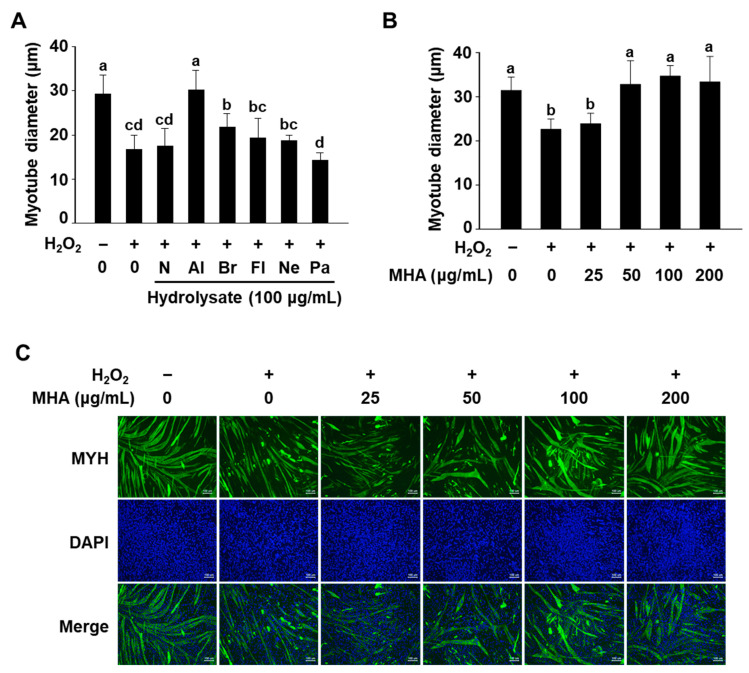
Effect of mackerel protein hydrolysates on myotube diameter in H_2_O_2_-treated C2C12 myotubes. (**A**,**B**) Differentiated C2C12 myotubes were treated with H_2_O_2_ (2 mM) in the presence of the indicated hydrolysate for 24 h, and the diameter of myotubes was measured. (**C**) Immunofluorescence staining of MYH (green); nuclei were counterstained with DAPI (blue). Scale bars = 100 μm (magnification ×100). Data are presented as mean ± SD (n ≥ 3), and different superscripts (a–d) are significantly different at *p* < 0.05 by Duncan’s multiple range test. N: no enzyme, Al: alcalase, Br: bromelain, Fl: flavourzyme, Ne: neutrase, Pa: papain.

Differentiation of C2C12 myotubes is characterized by cell fusion, leading to an increase in myotube number and diameter. In contrast, muscle atrophy results in protein degradation and myotube shrinkage [25,26,27]. To evaluate the protective effects of MPHs, differentiated C2C12 myotubes were pre-treated with 100 μg/mL of each MPH for 1 h, followed by H_2_O_2_ (2 mM) treatment for 24 h. Among the tested hydrolysates, only MHA (alcalase-derived hydrolysate) restored myotube diameter to near-normal levels, while bromelain hydrolysate showed only a slight improvement. Other MPHs did not show significant protective effects compared to the H_2_O_2_-only treated group (Figure 2A). Based on these results, MHA was identified as the most effective hydrolysate for protecting against myotube atrophy, and subsequent experiments were conducted using MHA.

Before evaluating the dose-dependent effects of MHA, its cytotoxicity in C2C12 myotubes was assessed. No toxicity was observed up to 200 μg/mL, but at 400 μg/mL, cell viability decreased by approximately 10% (Supplemental Figure S1). Therefore, MHA concentrations were set between 25 and 200 μg/mL for subsequent experiments. At 25 μg/mL, MHA showed no protective effect, whereas concentrations ≥50 μg/mL significantly restored myotube diameter (Figure 2B). However, there was no dose-dependent effect beyond 50 μg/mL, as myotube diameter was already restored to control levels at this concentration.

A hallmark of myotube atrophy is the decreased expression of muscle-specific proteins, such as myosin heavy chain (MYH), along with the upregulation of muscle protein degradation pathways, particularly through ubiquitin E3 ligases, including MAFbx and MuRF1. To investigate whether MHA could modulate these biomarkers, we assessed their expression levels in H_2_O_2_-treated C2C12 myotubes using immunofluorescence staining, Western blot, and quantitative PCR (qPCR).

First, immunofluorescence staining was performed to visualize MYH expression directly within the cells (Figure 2C). As expected, treatment with 2 mM H_2_O_2_ markedly reduced both MYH expression and myotube diameter compared to untreated controls, confirming the induction of atrophy. In contrast, the MHA-treated group showed MYH expression and myotube morphology comparable to the untreated control group, indicating a protective effect against H_2_O_2_-induced atrophy.

**Figure 3:**
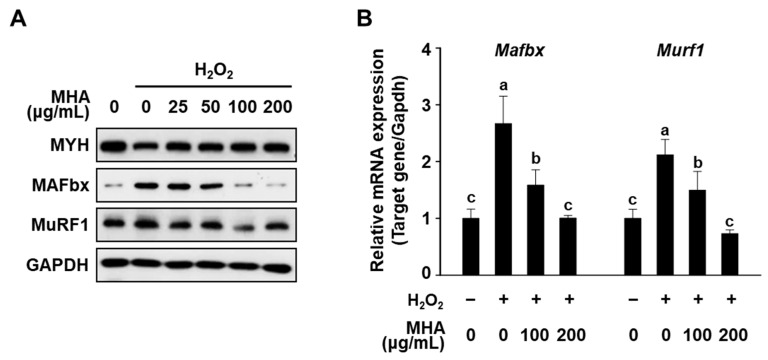
Effects of MHA on the expression of MYH, MAFbx, and MuRF1 in H_2_O_2_-treated C2C12 myotubes. Differentiated C2C12 myotubes were treated with H_2_O_2_ (2 mM) in the presence of MHA for 24 h (**A**) or 18 h (**B**). (**A**) Protein expression levels of MYH, MAFbx, and MuRF1 assessed by Western blot. (**B**) mRNA expression levels of the same genes were analyzed by qPCR, with Gapdh used as an internal control. Data are presented as mean ± SD (n ≥ 3), and different superscripts (a–c) are significantly different at *p* < 0.05 by Duncan’s multiple range test.

Western blot analysis was conducted to quantitatively assess the expression of MYH and the two ubiquitin E3 ligases, MAFbx and MuRF1 (Figure 3A). Consistent with the immunofluorescence results, H_2_O_2_ treatment alone led to decreased MYH protein levels and significant upregulation of MAFbx and MuRF1, confirming the atrophic phenotype. However, co-treatment with MHA significantly restored MYH levels while reducing MAFbx and MuRF1 expression, and MAFbx expression decreased in a dose-dependent manner with increasing MHA concentrations (25–200 μg/mL). Furthermore, qPCR analysis demonstrated that MHA significantly suppressed the mRNA expression of MAFbx and MuRF1 compared to the H_2_O_2_-only group (Figure 3B), further supporting the protein-level findings. These results suggest that MHA protects C2C12 myotubes from H_2_O_2_-induced atrophy by preserving muscle protein expression and downregulating key atrophy-related genes at both transcriptional and translational levels.

Interestingly, while the protective effect of MHA on myotube diameter plateaued at doses above 50 μg/mL, the expression of the atrophy-related gene MAFbx continued to decrease in a dose-dependent manner. This discrepancy may be attributed to differences in sensitivity and response kinetics between morphological and molecular endpoints. Myotube diameter reflects cumulative structural changes and may reach a physiological limit beyond which further recovery is not morphologically evident. In contrast, MAFbx responds more dynamically at the molecular level and may continue to be suppressed by higher doses of MHA even after morphological recovery has reached a plateau.

In addition to the well-characterized ubiquitin-proteasome system, recent evidence highlights a critical role for cysteine proteases, particularly calpain-1, in the early stages of skeletal muscle atrophy under oxidative stress conditions [28]. Knockdown of calpain-1 via siRNA significantly attenuated H_2_O_2_-induced myotube diameter loss and cleavage of its substrate αII-spectrin, indicating its essential role in myofilament protein release and early proteolytic signaling. Our study, while primarily focused on the modulation of ubiquitin ligases, observed a strong correlation between MHA treatment and the preservation of myotube morphology and MYH levels. These protective effects may extend beyond the transcriptional regulation of atrogenes, potentially involving inhibition of upstream proteolytic mechanisms such as calpain activation. Given that calpain-mediated myofibrillar protein release is a prerequisite for subsequent degradation by the proteasome, it is plausible that MHA may also interfere with calpain activity, either directly or through modulation of oxidative stress-induced calcium signaling, which is known to activate calpains.

Future studies will aim to examine whether MHA suppresses calpain-1 activity or its downstream proteolytic substrates during oxidative stress-induced atrophy. Additionally, assessing changes in intracellular Ca^2+^ levels and the expression of calpastatin (the endogenous calpain inhibitor) may help clarify MHAs role in modulating early proteolytic events. These investigations will provide deeper insight into MHAs anti-atrophic mechanisms, encompassing both transcriptional and non-transcriptional pathways involved in muscle protein degradation.

### 3.3. MHA Modulates Akt/FoxO and NF-κB Pathways in H_2_O_2_-Induced Myotube Atrophy

Akt (protein kinase B) is a central regulator of cell growth and survival and plays a pivotal role in the PI3K-Akt signaling pathway, which governs numerous cellular functions, including muscle protein homeostasis [29]. Akt is activated by various growth factors, such as insulin-like growth factor 1 (IGF-1), leading to the phosphorylation and inactivation of FoxO transcription factors. FoxO, when active, translocates into the nucleus and induces the expression of key ubiquitin E3 ligases, including MAFbx and MuRF1, which are responsible for muscle protein degradation. Conversely, activated Akt phosphorylates FoxO, preventing its nuclear localization and thus inhibiting the transcription of these atrophy-related genes. Therefore, the Akt/FoxO pathway is crucial in maintaining the balance between muscle protein synthesis and degradation.

In parallel, the NF-κB pathway—comprising transcription factors such as p65 (RelA); RelB; c-Rel; p50 (NFκB1); and p52 (NFκB2)—regulates various cellular processes; including inflammation and immune responses [30]. Typically, NF-κB exists in the cytoplasm bound to IκB, which keeps it inactive. Upon stimulation, IκB is phosphorylated and degraded, releasing NF-κB to translocate into the nucleus, where it promotes the transcription of pro-inflammatory genes. Beyond its role in inflammation, NF-κB is also implicated in muscle atrophy, particularly through the transcriptional activation of MuRF1, contributing to muscle protein degradation [31].

**Figure 4:**
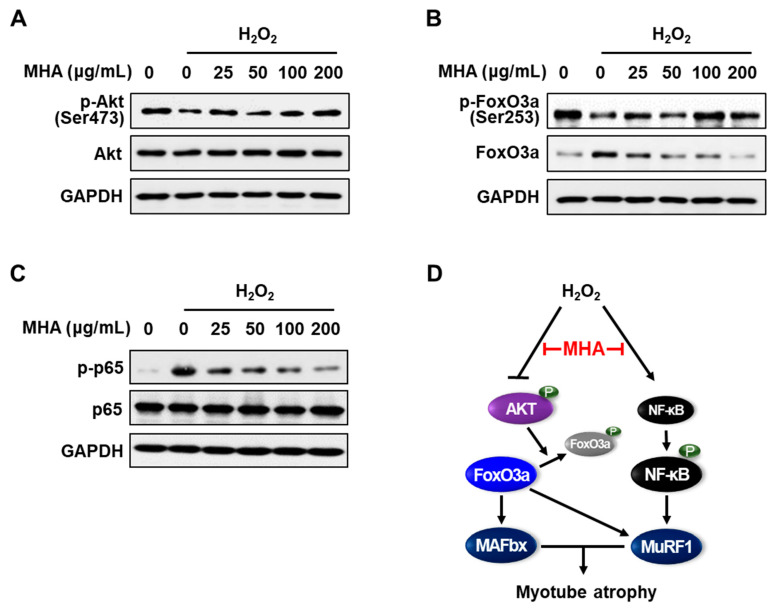
Effects of MHA on Akt/FoxO and NF-κB signaling pathways in H_2_O_2_-treated C2C12 myotubes. Differentiated C2C12 myotubes were treated with H_2_O_2_ (2 mM) in the presence of MHA for 12 h (**A**), 16 h (**B**), or 0.5 h (**C**). Whole-cell lysates were collected and analyzed by Western blot, with GAPDH used as a loading control. (**D**) Schematic diagram illustrating how MHA modulates key signaling pathways involved in oxidative stress-induced myotube atrophy.

To further elucidate the mechanism underlying the protective effects of MHA, we investigated how H_2_O_2_-induced atrophy affects the Akt/FoxO and NF-κB pathways and whether MHA modulates these signaling cascades. As shown in Figure 4, treatment with H_2_O_2_ alone resulted in a marked decrease in phosphorylated Akt (Ser473) and phosphorylated FoxO3a (Ser253), alongside a significant increase in phosphorylated NF-κB p65 (Ser536). This confirms that oxidative stress-induced atrophy follows the typical pattern of Akt inactivation, FoxO activation, and NF-κB activation, which collectively promote muscle protein breakdown.

However, co-treatment with MHA effectively reversed these effects. Specifically, MHA significantly restored Akt phosphorylation, particularly at concentrations ≥25 μg/mL, and increased FoxO3a phosphorylation to levels comparable to or higher than the untreated control across all concentrations (Figure 4A,B). Conversely, phosphorylation of NF-κB p65 was dramatically reduced starting at 25 μg/mL MHA, with a dose-dependent decrease observed up to 200 μg/mL (Figure 4C). These findings are consistent with the observed downregulation of MAFbx and MuRF1 expression at both the protein and mRNA levels (Figure 3), indicating that MHA exerts its anti-atrophic effects by activating the Akt/FoxO pathway and suppressing NF-κB signaling. Consequently, MHA inhibits the transcription of key ubiquitin E3 ligases, thereby preventing muscle protein degradation under oxidative stress conditions.

Several recent studies have demonstrated that protein hydrolysates derived from various food sources exert protective effects against muscle atrophy through modulation of protein degradation pathways [14,32,33,34,35,36]. For instance, spirulina protein hydrolysate (SPH) significantly alleviated dexamethasone-induced muscle atrophy in C2C12 myotubes by activating the Akt/FoxO3a pathway and downregulating the expression of key atrogenes, MuRF1 and MAFbx. Additionally, SPH promoted myogenic differentiation by upregulating MyoD1 and Myf5, suggesting dual functionality in both atrophy prevention and muscle regeneration. Similarly, yeast hydrolysate showed anti-atrophic effects in both in vitro and in vivo models, attenuating the expression of MuRF1 and FoxO3a and preserving muscle mass and strength in dexamethasone-treated mice.

In line with previous findings, our study demonstrated that MHA effectively restored myotube diameter and MYH expression in H_2_O_2_-induced C2C12 atrophy while significantly downregulating MuRF1 and MAFbx at both the mRNA and protein levels. Mechanistic analyses further revealed that these protective effects are mediated by regulating the ubiquitin-proteasome system, primarily via the Akt/FoxO and NF-κB signaling pathways. Collectively, these results highlight the potential of MHA as a promising functional ingredient for preventing muscle atrophy by modulating key catabolic signaling mechanisms. To further validate its efficacy and potential for practical application, future studies employing in vivo models will be necessary.

### 3.4. Antioxidant Activity of MHA Contributes to Its Protective Effects Against H_2_O_2_-Induced Myotube Atrophy

Oxidative stress and disrupted redox homeostasis are widely recognized as key factors contributing to muscle atrophy, particularly in H_2_O_2_-treated C2C12 myotubes [37,38]. In recent years, protein hydrolysates derived from various food sources have attracted attention for their potent antioxidant properties. These effects are largely attributed to the presence of low-molecular-weight peptides and free amino acids, which can directly scavenge reactive oxygen species (ROS) and neutralize free radicals. In addition to their direct antioxidant action, some hydrolysates have also been reported to enhance cellular antioxidant defense mechanisms by upregulating the expression of antioxidant-related genes in various cell models [15,39]. These dual actions suggest that protein hydrolysates hold promise as functional ingredients for combating oxidative stress-related muscle atrophy.

**Figure 5:**
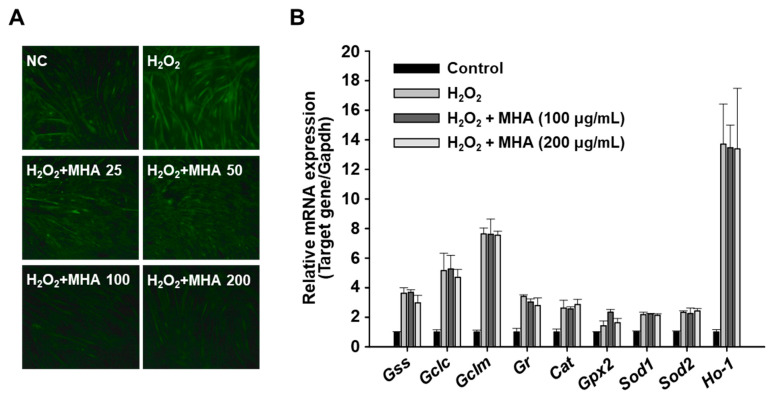
Effects of MHA on intracellular ROS levels and antioxidant gene expression in H_2_O_2_-treated C2C12 myotubes. Differentiated C2C12 myotubes were treated with H_2_O_2_ (2 mM) in the presence of MHA for 6 h (**A**) or 18 h (**B**). (**A**) Intracellular ROS levels were assessed using the fluorescent probe DCFH-DA (green; magnification ×100). (**B**) mRNA expression levels of antioxidant genes were analyzed by qPCR, with GAPDH used as an internal control. Data are presented as mean ± SD (n ≥ 3). Gss: glutathione synthetase, Gclc: glutamate-cysteine ligase, Gclm: glutamate-cysteine ligase modifier subunit, Gr: glutathione reductase, Cat: catalase, Gpx2: glutathione peroxidase 2, Sod: superoxide dismutase, Ho-1: heme oxygenase 1.

Based on this, we hypothesized that MHA might exhibit similar antioxidant properties, potentially contributing to its protective effects against H_2_O_2_-induced myotube atrophy. To investigate this, intracellular ROS levels were measured in C2C12 myotubes treated with H_2_O_2_ alone or in combination with MHA. As expected, H_2_O_2_ markedly elevated ROS levels, while co-treatment with MHA significantly reduced intracellular ROS (Figure 5A), suggesting that MHA mitigates oxidative stress in myotubes.

To further assess the antioxidant potential of MHA, we evaluated its direct ROS-scavenging activity against H_2_O_2_ and ABTS radicals. The results showed that MHA scavenged both ROS species in a dose-dependent manner, with RC_50_ values below 80 μg/mL (Table 1). Notably, these effective concentrations closely correspond to those at which MHA exerted protective effects on myotube diameter, supporting a direct link between the antioxidant and anti-atrophic properties of MHA.

We then examined whether MHA modulates the expression of antioxidant-related genes, glutathione synthetase (Gss), glutamate-cysteine ligase (Gclc), glutamate-cysteine ligase modifier subunit (Gclm), glutathione reductase (Gr), catalase (Cat), glutathione peroxidase 2 (Gpx2), superoxide dismutases (Sod1 and Sod2), and heme oxygenase 1 (Ho-1), in C2C12 myotubes using qPCR. Interestingly, MHA treatment did not significantly affect the expression levels of any antioxidant genes tested (Figure 5B), differing from previous reports where various protein hydrolysates enhanced antioxidant gene expression [15,39]. These results suggest that the antioxidant action of MHA in myotubes is not mediated by transcriptional regulation, but rather by direct ROS scavenging. This difference may be attributed to variations in peptide composition or cell-type-specific responses.

Furthermore, in line with previous reports highlighting oxidative stress as a key driver of muscle atrophy [37,38], our findings suggest that the antioxidant activity of MHA plays a crucial role in protecting C2C12 myotubes from H_2_O_2_-induced damage. A recent study on cinnamaldehyde demonstrated that reducing ROS levels can preserve myotube morphology and suppress proteolytic systems, including the ubiquitin-proteasome pathway, autophagy, and lysosomal proteases, while also maintaining the activity of antioxidant defense enzymes [38]. Although MHA did not significantly alter antioxidant gene expression, its direct ROS-scavenging capacity may contribute to cellular redox balance and the inhibition of proteolytic signaling involved in muscle degradation. Collectively, these results indicate that MHAs antioxidant action, particularly its direct neutralization of ROS, plays a significant role, at least in part, in mitigating oxidative stress-induced myotube atrophy.

### 3.5. Chemical and Nutritional Characteristics of MHA

Table 2 summarizes the general composition, mineral content, and heavy metal levels in MHA. On a dry matter basis, MHA consisted of 92.04% protein, 1.21% lipid, and 0.93% carbohydrates, indicating that protein is the primary component. Among the minerals analyzed, sodium, potassium, phosphorus, magnesium, and calcium were the most abundant, in descending order, with all other minerals detected at levels below 0.01%. In terms of heavy metal content, lead (0.002 mg/kg) and mercury (0.001 mg/kg) were detected at very low levels, while cadmium was not detected at all, confirming that MHA complies well below the regulatory safety limits for food-grade materials.

Amino acids are the fundamental building blocks of proteins and play critical roles in muscle growth, repair, and metabolism. Particularly, branched-chain amino acids (BCAAs) such as leucine, isoleucine, and valine have been shown to stimulate muscle protein synthesis and mitigate muscle atrophy in both animal models and clinical studies [40,41,42].

As shown in Table 3, a total of 694.02 mg of amino acids was detected per gram of MHA. The most abundant amino acids were glutamic acid (15.75%), aspartic acid (10.05%), and lysine (9.04%). BCAAs accounted for 16.66%, aromatic amino acids (AAAs) for 5.54%, and hydrophobic amino acids made up 38.65% of total amino acids. This profile reflects the typical characteristics of animal-derived proteins, which generally exhibit higher proportions of BCAAs and essential amino acids (EAAs) compared to plant-derived proteins [43]. Notably, leucine and lysine contents in MHA contribute to this EAA-rich profile, further aligning MHA with other functional animal protein hydrolysates. A recent study using dexamethasone-treated C2C12 myotubes showed that beef protein hydrolysate, with a similar BCAA/EAA composition, exerted anti-atrophic effects at 25–100 μg/mL—a concentration range comparable to MHA [14].

In terms of free amino acids, histidine (15.82%) and β-aminoisobutyric acid (12.93%) were the most abundant, followed by AAAs (7.24%), BCAAs (8.20%), and hydrophobic amino acids (22.27%) (Supplemental Table S2). Although present, the levels of free amino acids were relatively lower than those observed in total amino acid analysis. While both BCAAs and arginine have been shown to suppress the expression of MAFbx and MuRF1 at concentrations around 5 mM in C2C12 cells, and even at 1 mM in the case of leucine [44], the concentrations of these free amino acids in MHA are substantially lower than the effective doses reported in prior studies. Therefore, the protective effect of MHA on muscle atrophy cannot be attributed solely to its amino acid content.

Given the limited role of free amino acids, the bioactivity of MHA is likely influenced by its content of short-chain peptides. Molecular weight analysis using aqueous gel permeation chromatography (GPC) revealed that MHA primarily consists of components <4000 Da, with prominent peaks at 2344, 595, and 247 Da (Supplemental Figure S2). These results are consistent with SDS-PAGE analysis (Figure 1B), which also confirmed the formation of low-molecular-weight peptides via alcalase hydrolysis.

Recent studies have demonstrated a wide range of physiological functions of short-chain peptides derived from food proteins, including antioxidant, anti-atrophic, antimicrobial, antihypertensive, and lipid-regulating activities [14,20,22,24,45,46]. For example, Cho and Lee (2020) [15] isolated LE and AKKHKE peptides from alcalase-treated mealworm hydrolysates that showed both antioxidant and hepatoprotective effects. In another study, Hur et al. [14] reported that two short peptides, AFRSSTKK and GAGAAGAPAGGA, protected skeletal muscle from atrophy. Similarly, zein-derived peptides were recently shown to promote myoblast proliferation and increase protein synthesis via mTORC1 and mTORC2 activation in C2C12 cells, further supporting the role of dietary peptides in modulating muscle-regulatory pathways [47]. These findings suggest that the protective effect of MHA on myotubes may result not only from its amino acid composition but also from the synergistic action of various bioactive short peptides. Ongoing studies are currently focused on profiling these peptides, isolating active sequences, and identifying their specific mechanisms involved in muscle cell protection.

## 4. Conclusions

This study demonstrated that alcalase-derived mackerel protein hydrolysate (MHA) effectively protects against oxidative stress-induced myotube atrophy in H_2_O_2_-treated C2C12 cells. Among various enzyme-treated hydrolysates, MHA showed the strongest ability to preserve myotube diameter, suppress atrophy-related genes (MAFbx and MuRF1), and restore MYH protein levels. Mechanistic analysis revealed that MHA acts by activating the Akt/FoxO pathway and inhibiting NF-κB signaling, thereby reducing muscle protein degradation. MHA also significantly reduced intracellular ROS and exhibited direct antioxidant activity, supporting its role in oxidative stress protection. Amino acid and molecular weight profiling showed that MHA is rich in essential amino acids—notably BCAAs and lysine—and contains a high proportion of low-molecular-weight peptides, which likely contribute to its bioactivity. While the free amino acid content alone was insufficient to account for the protective effects, short-chain peptides appear to play a key synergistic role. Overall, MHA is a promising food-derived functional ingredient with anti-atrophic and antioxidant properties, offering potential for use in the prevention or management of age-related muscle loss (sarcopenia). Further studies are underway to isolate and characterize the specific bioactive peptides responsible for these effects.

## Figures and Tables

**Table 1:** Direct scavenging effects of MHA on H_2_O_2_ and ABTS radicals.

	H_2_O_2_		ABTS	
	RC_50_ (μg/mL) ^(1)^	TEAC (μg TE/mg) ^(3)^	RC_50_ (μg/mL) ^(2)^	TEAC (μg TE/mg) ^(3)^
MHA	63.24 ± 1.08 ^(4)^	96.32 ± 0.82	78.28 ± 0.96	58.72 ± 0.75
Trolox	6.31 ± 0.09		5.20 ± 0.02	

^(1)^ Concentration required for 50% reduction of H_2_O_2_ at 10 min after starting the reaction. ^(2)^ Concentration required for 50% reduction of ABTS^+^ at 1 min after starting the reaction. ^(3)^ Trolox equivalent antioxidant capacity. ^(4)^ Each value is mean ± SD (n ≥ 3).

**Table 2:** Nutrient and heavy metal contents of MHA.

	Component	Concentration (mg/g)	Percentage (% Dry Basis)
Macronutrients	Carbohydrate	6.8	0.93
	Protein	672.0	92.04
	Lipid	8.8	1.21
Minerals	Calcium	2.1760	0.30
	Sodium	15.0532	2.06
	Magnesium	2.6874	0.37
	Iron	0.0034	<0.01
	Copper	0.0052	<0.01
	Phosphorus	8.3064	1.14
	Zinc	0.0140	<0.01
	Selenium	0.0034	<0.01
	Potassium	14.2938	1.96
	Iodine	0.0006	<0.01
	Manganese	0.0002	<0.01
Heavy metals (mg/kg)	Lead	0.0020	<0.01
	Cadmium	− ^(1)^	<0.01
	Mercury	0.0010	<0.01
Total		730.14	100
Calorie (Kcal/g)		2.794	

^(1)^ Not detected.

**Table 3:** Amino acid composition of MHA.

Amino Acid	mg/g	%
Alanine	48.87	7.04
Arginine	43.17	6.22
Aspartic acid	69.78	10.05
Cystine	5.47	0.79
Glutamic acid	109.29	15.75
Glycine	47.97	6.91
Histidine	39.36	5.67
Isoleucine	27.49	3.96
Leucine	54.24	7.82
Lysine	62.75	9.04
Methionine	16.99	2.45
Phenylalanine	23.09	3.33
Proline	27.22	3.92
Serine	33.66	4.85
Threonine	35.48	5.11
Tryptophan	<0.01	<0.01
Tyrosine	15.33	2.21
Valine	33.87	4.88
Total amino acid	694.02	100
AAA	38.42	5.54
BCAA	115.61	16.66
HAA	268.22	38.65
EAA	293.27	42.24

AAA: aromatic amino acids (phenylalanine and tyrosine); BCAA: branched-chain amino acids (isoleucine, leucine, and valine); HAA: hydrophobic amino acids (glycine, alanine, proline, cystine, valine, isoleucine, leucine, and phenylalanine); EAA: essential amino acid (histidine, isoleucine, leucine, lysine, methionine, phenylalanine, threonine, tryptophan, and valine).

## Data Availability

The original contributions presented in this study are included in the article/Supplementary Materials. Further inquiries can be directed to the corresponding author.

## Supplementary Materials

The following supporting information can be downloaded at: https://www.mdpi.com/article/10.3390/foods14142430/s1, Supplemental Materials and Methods, Table S1. Production yield of mackerel protein hydrolysate by various enzymes. Table S2. Free amino acid composition of MHA. Table S3. List of antibodies used in this study. Table S4. Sequence of the primers used for qPCR. Figure S1. Effect of MHA on cell viability in H2O2-treated C2C12 myotubes. Figure S2. GPC chromatogram of MHA.
