# Supplementary material for: Protective Effects of Mackerel Protein Hydrolysates Against Oxidative Stress-Induced Atrophy in C2C12 Myotubes
Source: Foods. 2025 Jul 10;14(14):2430. doi: 10.3390/foods14142430 (PMC12294637; doi:10.3390/foods14142430)
Supplement: Supplementary file 1 [file foods-14-02430-s001.zip › foods-3704364-supplementary.pdf]

SUPPLEMENTAL MATERIALS

Materials and Methods

4 Tables

2 Figures

Supplemental Materials and Methods

Cell viability assay

Cell viability was assessed using the MTT assay. Differentiated C2C12 cells were treated with MPH at various concentrations 1 h before exposure to 2 mM H_2O_2 for 24 h. After treatment, 50 µL of MTT reagent (2.5 mg/mL) was added to each well and incubated for 4 h. The supernatant was removed, and 500 µL of DMSO was added to dissolve the formazan crystals. Absorbance was measured at 550 nm using a microplate spectrophotometer. Cell viability was expressed as a percentage relative to the control group.

Western blot analysis

C2C12 cells were lysed using 400 µL of RIPA buffer, and lysates were centrifuged at 13,000 ×g for 15 min. Proteins (5–30 µg per lane) were separated on 8–15% SDS-PAGE gels and transferred onto PVDF membranes. After blocking with 5% skim milk, membranes were incubated overnight at 4°C with primary antibodies (diluted 1:1,000 in 5% BSA solution). After washing with PBST, HRP-conjugated secondary antibodies were applied (1:3,000 dilution), followed by detection using an ECL detection kit and visualization on X-ray film. The antibodies used are listed in Supplementary Table S3.

Analysis of mRNA expression using quantitative real-time PCR

After treating C2C12 cells, the culture medium was removed, and cells were washed twice with PBS. TRIzol reagent (400 µL) was added, and lysates were transferred to 1.5 mL microcentrifuge tubes. After adding 80 µL of chloroform, samples were vortexed for 30 sec, incubated at room temperature for 15 min, and centrifuged at 13,000 ×g, 4°C for 15 min. The supernatant was transferred to a new tube, mixed with 200 µL of isopropyl alcohol, incubated

on ice for 10 min, and centrifuged again. The RNA pellet was washed with 70% ethanol (300 μL), air-dried, and dissolved in DEPC-treated water at 65°C for 10 min. RNA concentration and purity were measured at 260/280 nm using a spectrophotometer. For cDNA synthesis, 1 μg of total RNA was mixed with 1 μL of oligo(dT) primer and adjusted to 12 μL with DEPC-treated water. After incubation at 65°C for 5 min, 1 μL of DEPC-treated water, 4 μL of 5× reaction buffer, 2 μL of 10 mM dNTP mix, 0.5 μL of RNase inhibitor (20 U/μL), and 0.5 μL of reverse transcriptase were added. The reaction was performed at 42°C for 60 min, followed by inactivation at 70°C for 5 min. For quantitative real-time PCR (qPCR), 2 μL of cDNA was combined with 5 μL of SYBR Green I dye, 2.5 μL of DEPC-treated water, and 0.5 μL of each primer (Supplementary Table S4). The reaction conditions were initial denaturation at 95°C for 30 sec, followed by 40 cycles of 95°C for 5 sec and 60°C for 30 sec.

Proximate composition and mineral/heavy metal analysis

The crude protein and crude fat contents of mackerel powder were determined according to the Korean Food Code (sections 2.1.3 and 2.1.5), which are based on AOAC Official Methods 984.13 and 920.39, respectively. Carbohydrate content was calculated by difference using the formula:

Carbohydrates (%) = 100 – (moisture + protein + fat + ash).

Mineral contents, including calcium (Ca), sodium (Na), magnesium (Mg), iron (Fe), copper (Cu), phosphorus (P), zinc (Zn), potassium (K), and manganese (Mn), were measured by inductively coupled plasma–optical emission spectrometry (ICP-OES; Avio 200, PerkinElmer, USA) following microwave-assisted acid digestion (MARS6, CEM, USA), in accordance with AOAC Official Method 2011.14 and the Korean Food Code (section 2.2.1). Iodine content was analyzed using inductively coupled plasma–mass spectrometry (ICP-MS; NexION 300D, PerkinElmer, USA) after tetramethylammonium hydroxide (TMAH) extraction,

based on FDA Method 4.13 (2017). Selenium (Se) was determined using ICP-MS according to AOAC Official Method 2015.06 and the Korean Food Code (section 2.2.1). Heavy metals, including lead (Pb) and cadmium (Cd), were quantified by ICP-MS following AOAC Official Method 2015.01 and the Korean Food Code (section 9.1.2). Mercury (Hg) content was measured using a mercury analyzer based on cold vapor atomic absorption spectrometry, according to AOAC Official Method 974.14 and the Korean Food Code (section 9.1.6).

Gel permeation chromatography (GPC) analysis

GPC analysis was performed using an Alliance e2695 instrument (Waters, USA) equipped with an RI-detector. Waters Ultrahydrogel Linear, Waters Ultrahydrogel 500, Waters Ultrahydrogel 250, and Waters Ultrahydrogel 120 were used as the analytical columns and the solvent (0.02 N $NaNO_3$). The column temperature was maintained at 35℃. The analysis was performed at a flow rate of 0.8 mL/min.

Supplemental Table S1. Production yield of mackerel protein hydrolysate by various enzymes

Enzymes	Production yield of hydrolysate (%)[1)]
No enzyme	$12.25\pm0.01^{d2)}$
Alcalase	47.18 ± 0.00^{a}
Bromelain	39.67 ± 0.00^{b}
Flavourzyme	36.34 ± 0.10^{bc}
Neutrase	29.26 ± 0.01^{c}
Papain	19.43 ± 0.01^{d}

[1)]Yield (%) = [total solid content of protein hydrolysate – total solid content of blank (without substrate)]/total substrate content × 100

[2)]Data are presented as mean±SD (n≥3) and different superscripts (a-d) are significantly different at $p<0.05$ by Duncan's multiple range test.

Supplemental Table S2. Free amino acid composition of MHA

Amino acid	mg/g	(%)
Alanine	11.93	4.61
Anserine	15.33	5.93
Arginine	4.32	1.67
Aspartic acid	3.24	1.26
Carnosine	6.80	2.63
Cystine	9.58	3.71
Glutamic acid	7.87	3.04
Glycine	3.43	1.33
Histidine	40.89	15.82
Hydroxy proline	0.39	0.15
Hydroxylysine	1.42	0.55
Isoleucine	7.90	3.06
Leucine	7.32	2.83
Lysine	6.13	2.37
Methionine	5.29	2.05
3-Methylhistidine	2.53	0.98
Ornithine	2.33	0.90
Phenylalanine	8.17	3.16
Phosphoserine	2.15	0.83
Proline	3.27	1.27
Serine	2.85	1.10
Taurine	20.12	7.78
Threonine	3.65	1.41
Tyrosine	10.55	4.08
Urea	23.03	8.91

(continued on next page)

Supplementary Table 2 (continued)

Amino acid	mg/g	(%)
Valine	5.97	2.31
β-Alanine	3.18	1.23
β-Amino isobutyric acid	33.42	12.93
γ-Amino-n-butyric acid	5.39	2.09
Total free amino acid	258.45	100
AAA	18.71	7.24
BCAA	21.19	8.20
HAA	57.56	22.27
EAA	85.32	33.01

AAA: aromatic amino acids (phenylalanine and tyrosine); BCAA: branched-chain amino acids (isoleucine, leucine, and valine); HAA: hydrophobic amino acids (glycine, alanine, proline, cystine, valine, isoleucine, leucine, and phenylalanine); EAA: essential amino acids (histidine, isoleucine, leucine, lysine, methionine, phenylalanine, threonine, tryptophan, and valine).

Supplemental Table S3. List of antibodies used in this study

Antibody	Vander	No.	Dilution ratio
MYH	Santa cruz	sc-376157	1:200 (IF)<br>1:20000 (WB)
MAFbx	Santa cruz	sc-166806	1:1000
MuRF1	Santa cruz	sc-514767	1:300
Akt	Cell signaling	9272	1:1000
p-Akt	Cell signaling	9271	1:1000
FoxO3a	Cell signaling	12829	1:1000
p-FoxO3a	Cell signaling	9466	1:1000
p65	Cell signaling	4764	1:1000
p-p65	Cell signaling	3033	1:1000
GAPDH	Cell signaling	2118	1:3000
Anti-rabbit IgG-HRP	Cell signaling	7074	1:3000
Anti-mouse IgG-HRP	Cell signaling	7076	1:3000
Goat Anti mouse IgG<br>H&L (Alexa Fluor 488)	Abcam	ab 150113	1:200

Supplemental Table S4. Sequence of the primers used for qPCR

Target gene	Primer sequence	
Gapdh	Sense	5’-GTATGACTCCACTCACGGCA-3’
	Antisense	5’-GGTCTCGCTCCTGGAAGATG-3’
Tbp	Sense	5`-GTGAAGGGTACAAGGGGGTG-3`
	Antisense	5`-ACATCTCAGCAACCCACACA-3`
Mafbx	Sense	5’-CTCTGCTGTGAGTGCCACAT-3’
	Antisense	5’-CAATGAGCCTGGGTACCACT-3’
Murf1	Sense	5’-TGGAAACGCTATGGAGAACC-3’
	Antisense	5’-AACGACCTCCAGACATGGAC-3’
Gss	Sense	5’-GAAGCAGCTCGAAGAACTGG-3’
	Antisense	5’-AGCACTGGGTACTGGTGAGG-3’
Gclc	Sense	5’-CTGCACATCTACCACGCAGT-3’
	Antisense	5’-GTCTCAAGAACATCGCCTCC-3’
Gclm	Sense	5’-CCAAAACATCTGGAAACTCCC-3’
	Antisense	5’-CGGGAACCTGCTCAACTG-3’
Gr	Sense	5’-ATCGTGCATGAATTCCGAGT-3’
	Antisense	5’-GGTGGTGGAGAGTCACAAGC-3’
Cat	Sense	5’-CCCGCGGTCATGATATTAAGT-3’
	Antisense	5’-GATGAAGCAGTGGAAGGAGC-3’
Gpx2	Sense	5’-AGGTCGGACATACTTGAGGC-3’
	Antisense	5’-GGTAGTTCTCGGCTTCCCTT-3’
Sod1	Sense	5’-ACCATCCACTTCGAGCAGAA-3’
	Antisense	5’-AAAATGAGGTCCTGCACTGG-3’
Sod2	Sense	5’-AACTCAGGTCGCTCTTCAGC-3’
	Antisense	5’-GCTTGATAGCCTCCAGCAAC-3’
HO-1	Sense	5’-ACAACCAGTGAGTGGAGCCT-3’
	Antisense	5’-TCAAGGCCTCAGACAAATCC-3’

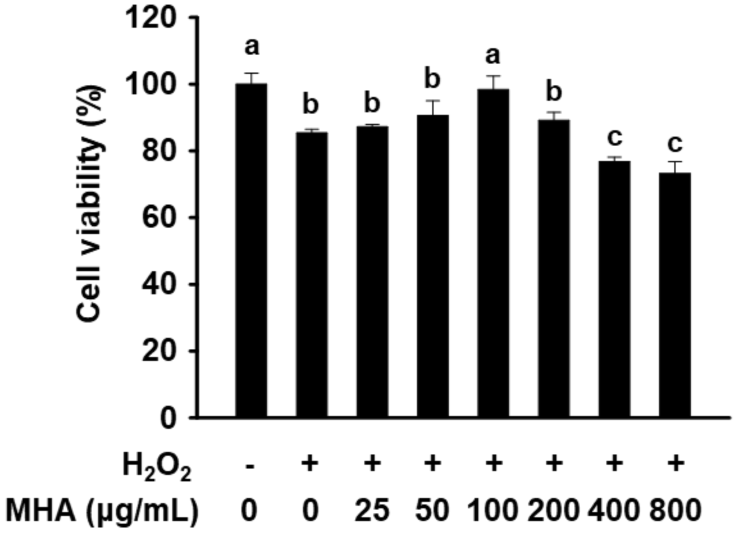

Supplemental Figure S1. Effect of MHA on cell viability in H_2O_2-treated C2C12 myotubes. Differentiated C2C12 myotubes were treated with H_2O_2 (2 mM) in the presence of the indicated concentration of MHA for 24h, and cell viability was determined by a WST-8 assay. All results are expressed as means±SD ($n \geq 3$), and different letters (a-c) are significantly different at $p<0.05$ by Duncan's multiple range test.

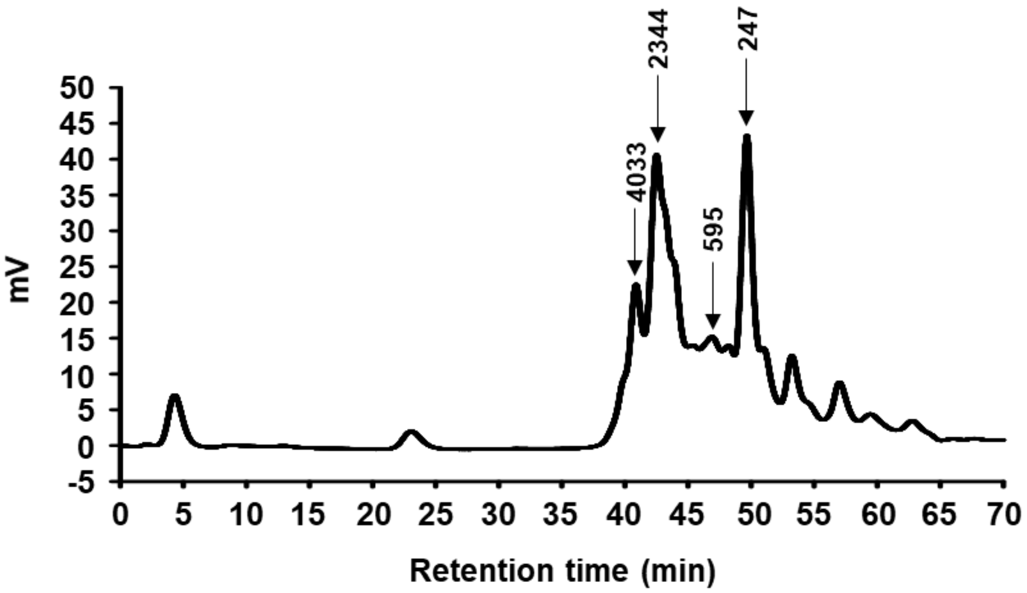

Supplemental Figure S2. GPC chromatogram of MHA.
